# Biotherapy Using Probiotics as Therapeutic Agents to Restore the Gut Microbiota to Relieve Gastrointestinal Tract Inflammation, IBD, IBS and Prevent Induction of Cancer

**DOI:** 10.3390/ijms24065748

**Published:** 2023-03-17

**Authors:** Divakar Dahiya, Poonam Singh Nigam

**Affiliations:** 1 Wexham Park Hospital, Wexham Street, Slough SL2 4HL, UK; 2 Biomedical Sciences Research Institute, Ulster University, Coleraine BT52 1SA, UK

**Keywords:** gut, microbiota, inflammation, probiotics, lactic acid bacteria, exopolysaccharide, short-chain fatty acids, IBS, IBD

## Abstract

The gut microbiota is composed of several microbial strains with diverse and variable compositions in both healthy and sick people. An undisturbed gut microbiota needs to be sustained in order to perform all physiological, metabolic, and immune functions in a normal way to prevent the development of diseases. This article has reviewed the published information on the issue of disruption of the balance of the gut microbiota. This disruption could be for many reasons, such as microbial infection in the gastrointestinal tract, food poisoning, diarrhoea, chemotherapy, malnutrition, lifestyle, and ageing. If this disruption is not restored to normal, it might cause dysbiosis. Eventually, a gut microbiota interrupted by dysbiosis might initiate several health issues, such as inflammation of the gastrointestinal tract, the induction of cancer, and the progression of a variety of diseases such as irritable bowel syndrome and inflammatory bowel disease. This review concluded that biotherapy is a natural way of using probiotic products, whether in form of food, beverages, or supplements, to restore the gut microbiota disrupted by dysbiosis. Metabolites secreted by the ingested probiotics help to relieve gastrointestinal tract inflammation and can avoid the induction of cancer.

## 1. Introduction

Microorganisms, including bacteria, fungi, viruses, and protozoa, inhabit the human system and affect various aspects of host physiology, such as metabolism, development of the immune system, and general well-being. The gut microflora, gut microbiota, or gut microbiome are commonly used terms for microbial strains that exist in the gastrointestinal tract. In healthy individuals, the gut is the main site for the survival of normal microbiota. A healthy, balanced microbiota in the gut is represented by several strains of non-pathogenic bacteria. Its composition and diversity is usually different at different stages of the human life, and it normally fluctuates from healthy individuals to persons suffering from different ailments, and also changes during and after chemotherapy using prescribed oral antibiotics [1]. The gut microbiome has an important role to play in the general health of a person through the regulation of host physiology [2].

Metabolites produced by strains constituting the normal gut microbiota induce beneficial immunological and cellular pathways to eliminate opportunistic strains of microorganisms which could be potential pathogens [3]. The metabolites of the gut microbiota know when to trigger an immune response to prevent ailments such as the inflammation of the gastrointestinal tract and cancer [4]. A healthy relationship between gut flora and the host is commensal, co-exists harmlessly, and is mutualistic. The ecology of the gut microbiota is different in separate parts of the gastrointestinal tract. Compared to a small number of bacteria generally present in the small intestine, the highest microbial population is found inside the large intestine. The dominant strains of bacteria isolated from the human gut have been identified as belonging to five major groups, including Firmicutes, Bacteroidetes, Actinobacteria, Proteobacteria, and Verrucomicrobia. Over 99% of the bacterial population present in the gut are anaerobes [1,5].

## 2. Activities of Balanced and Disturbed Gut Microbiota

An individual’s microbiota in its balanced state plays an essential role in sustaining health, and it also contributes to the development of health disorders and ailments if it is disturbed or imbalanced [6,7]. The gut microbiota supports the immune response of the host to assist chemo-therapeutic drugs being used for treatment. In a healthy host, the commensal microbiota stimulates inflammasomes that maintain the host’s cellular and gut integrity. In particular, the gut microbiota could inhibit the progression of a tumour via the activity of inflammasomes [8].

**Figure 1:**
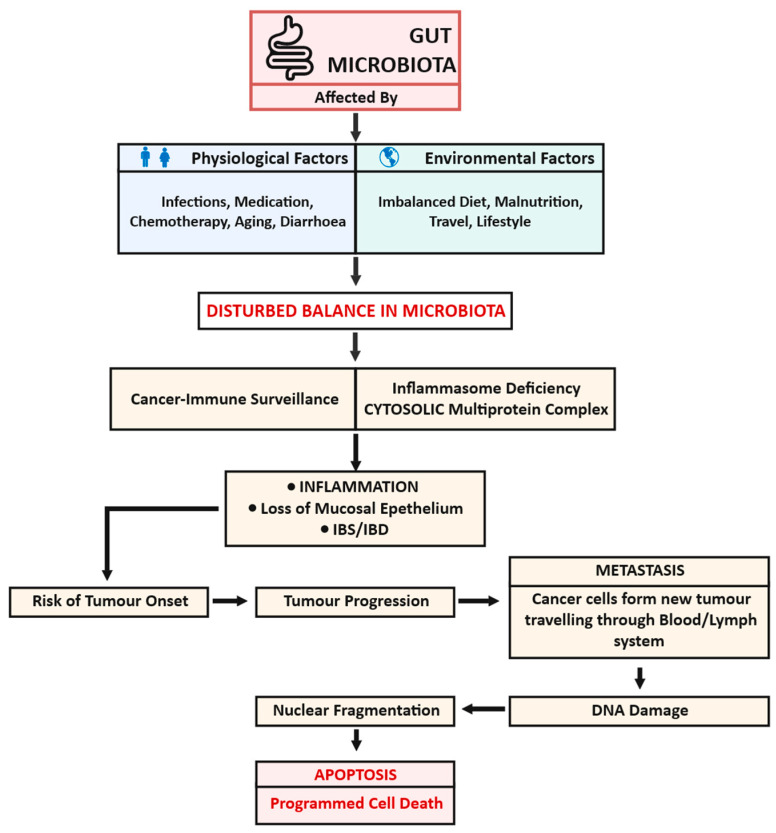
Variations in gut microbiota and their effect on the development and progression of health issues.

An imbalance in the healthy gut microbiota can cause the condition of dysbiosis, which can occur due to several factors such as bacterial infections [9]. Changes in the nature and function of gut microbiota are triggered by many reasons, such as an imbalanced diet, malnutrition, environmental factors, hygiene, immuno-compromised health conditions, gut infections, diarrhoea, and medication in the form of short- or long-term chemotherapy [10]. The variations in gut microbiota and their effect on the development and progression of health issues are summarized in Figure 1.

Any alteration in the composition of the gut microbes influences the tumour microbiota and the tumour microenvironment, thus affecting the progression of cancer and consequently causing DNA damage, nuclear fragmentation, and programmed cell death. Metastasis can also occur due to the movement of cancer cells from their site of development through the blood or lymph system, causing a new tumour at another site [6,7,8].

## 3. Inflammatory Action of Disturbed Gut Microbiota

A disorder in the healthy gut microbiome also disturbs the physical and microbial barriers of the gastrointestinal tract. Hence, any changes in the barrier’s integrity can have an effect on intestinal permeability, which in due course might advance the condition of inflammation and systemic disease [11,12]. Inflammatory bowel disease (IBD) is a term for two conditions, including Crohn’s disease and ulcerative colitis, that is characterized by a chronic inflammation of the gastrointestinal tract. A delay in the treatment of gut inflammation results in damage to the tract. The occurrence of IBD is often correlated with the condition of dysbiosis, which is accompanied by a high buildup of bacteria capable of managing oxidative stress, resulting in a considerable increase in the Enterobacteriaceae family of bacteria. Irritable bowel syndrome (IBS) can develop under the condition of dysbiosis caused after a continued period of diarrhoea caused by bacterial infections and gastroenteritis. IBS might also be associated with bacterial overgrowth in the intestines. An increased number of bacteria in the small intestine is referred to as a SIBO (small intestinal bacterial overgrowth). Inflammation affecting the epithelial barrier, abnormalities in gut motility, improper functioning of the immune system, and an abnormal population of bacteria cause symptoms of IBS [13].

Toxins from *Bacteroides fragilis* have been reported as coordinators of a pro-carcinogenic inflammatory cascade via the targeting colonic epithelial cells. Researchers have reported that toxins induce a pro-carcinogenic signalling relay from the colonic epithelial cells to a mucosal Th17 response. This results in NFκB activation, triggering myeloid cell-dependent distal colon tumorigenesis [14]. Though the role of the microbiome has been recognized in sustaining gut health, the precise mechanism of multi-fold functioning is still under investigation to understand the contribution of microbial factors in inhibition of cancer development and in the biotherapy of cancer. The mechanism of biotherapy is not as transparent as is the case with well-established chemo- and radio-therapies. However, it is understood that inflammation caused by some strains participates in the mechanism of carcinogenesis. It is reported that the metabolites secreted by some bacterial strains directly contribute to the action of colon cancer [15]. Some of these microbial metabolites act as toxins that can cooperate in several signalling pathways which regulate cancer-related biological processes, including cell propagation, cell growth cycle, differentiation, and programmed cell death (Figure 1) [16].

Chemotherapy using broad-spectrum antibiotics disrupts the gut microbiota and reduces the synthesis of intestinal short-chain fatty acids (SCFAs) by gut bacteria. The antibiotic-induced dysregulation of intestinal T-cell immunity has been reported as one of the mechanisms involved in the promotion of macrophage hyper-activation and the Th1 pro-inflammatory response [17]. Dysbiosis could also be due to repeated courses of antibiotic treatment in some patients. A disturbed gut microbiota with prolonged dysbiosis is correlated with pathogenicity, which may initiate tumorigenesis and the onset of several types of cancers [18,19].

The damaging effect of an imbalanced gut microbiota could initiate an increased risk of diarrhoea and gastrointestinal disorders. Uncontrolled dysbiosis may lead to colon cancer, irritable bowel syndrome, and inflammatory bowel disease by weakening the immune system and developing inflammation in parts of the gut. García-Castillo et al. published a very interesting study on microbiota dysbiosis, to be considered as a new piece in the puzzle for the understanding of carcinogenesis [20]. Zhang et al. have recently reported that any alteration in gastric microbiota is correlated with precancerous stages in gastric cancers [21]. Sun et al. studied the influence of gut microbiota on epigenetics in colorectal cancer [22]. The role of a disturbed gut microbiota on epigenetic modification and colorectal cancer has been discussed by Rezasoltani et al. [23].

## 4. Anti-Inflammatory Activities of Probiotics

Although the imbalance of the gut microbiota could be a reason for the beginning of inflammation and cancer, it can still be manipulated for the prevention of health disorders. It is claimed that several characterized strains of bacteria and yeast with probiotic characteristics may be used as biotherapeutic agents to re-establish the lost beneficial microbial population in the gastrointestinal tract [24,25].

The consumption of functional foods provides a supply of probiotic strains to the digestive system, which stimulates a microbial balance in the gastrointestinal tract of the host. Researchers have studied the effects of probiotic cultures and their beneficial role in the prevention of intestinal disorder conditions and the protection against diseases such as cancer, as well as in the activation of immune function, reduction of symptoms of IBS, and several other benefits to the host [26,27]. The secretion of bioactive molecules synthesized by probiotic strains triggers a strong immune response, which is a cellular molecular mechanism to inhibit the development of cancers and assists in the elimination of initial-stage cancers [28].

An active probiotic microbiota implements several biological effects through different mechanisms, such as competing for nutrients principally so they are sustained in the gut. Through this mechanism, the adhesion of pathogenic microorganisms to the epithelial cells in the gut is hindered. Furthermore, probiotics produce antagonistic compounds such as short-chain fatty acids (SCFA), bacteriocins, and organic acids that inhibit the pathogen’s growth and also obstruct the colonization of opportunistic microorganisms [4,6,8]. The association of fungi with the development and progression of carcinogenesis in the human body has been recently reviewed by Huët et al. [29]. The modulatory effects of the gut microbiota in anti-cancer therapy have been explored by Li et al. [30]. Probiotics have health-promoting activities through the synthesis of metabolites, which, as mentioned above, are mainly SCFA and organic acids, in the regulation of the immune system of the host. Plaza-Diaz et al. have reported the action of probiotics as stimulating the immune-globulin production, increasing the cytotoxicity of natural killer cells, and modulating cytokine secretion [31].

## 5. Biotherapy Using Probiotics

Antibiotic therapy is a common practice of chemotherapy for the treatment of microbial infections. These chemicals have the property of being anti to biotics targeting microorganisms. Antibiotics are used to kill infectious organisms and are delivered orally over a prescribed period of time; however, in such chemotherapy, the normal gut microbiota of patients is also adversely affected. As a result, after the prescribed course of antibiotic treatment, the loss of a balanced gut microbiota might cause the initiation of diarrhoea in some patients. Through a remedial therapy, the intake of fermented foods containing live probiotic strains of lactic acid bacteria, or alternatively, commercially available probiotic products, may prevent the disruption in the balance of gastrointestinal microbiota during and after antibiotic therapy by re-establishing the normal microbiota [32,33,34].

The strains resident in gut and their metabolites such as SCFAs affect the host’s immune response; hence, an imbalanced flora could be restored to its normal state by probiotic dietary practices or by taking commercial supplements available in health shops. Biotherapy of dysbiosis is beneficial for maintaining an optimal microbiota composition and benefitting from its anti-inflammatory activity. In addition, to prevent the condition of dysbiosis during chemotherapy treatment of gastric infections and acute diarrhoea, more pathogen-specific antibiotics with a narrow range of activity must be prescribed by health practitioners. It is also important to develop novel anti-bacterial strategies such as nano-medicine to aid in preserving eubiosis, an essential microbial balance within the gastrointestinal system [35]. As an alternative to broad-spectrum antibiotic therapy, the coupling of radiofrequencies with magnetic nanoparticle treatment has been suggested as an alternative physical-antibacterial strategy to treat multiple drug-resistant bacteria [36].

A probiotic strain of *Lacticaseibacillus paracasei* EPS DA-BACS capable of producing sufficient exopolysaccharides (EPSs) has been isolated from faeces samples of healthy humans. The probiotic properties, structure, and prebiotic activity of the EPS produced by this isolated bacterium were examined. EPSs synthesized by *L. paracasei* EPS DA-BACS had a ropy phenotype, which is known to have potential health benefits and is identified as a loosely cell-bounded glucomannan-type EPS. The molecular structure of EPSs promotes acid tolerance in probiotic strains, which is useful in the protection of epithelial cells under gastrointestinal stress [37]. Researchers have reported that *L. paracasei* EPS DA-BACS possesses no antibiotic resistance genes or virulence factors. Therefore, this strain exhibited anti-inflammatory and antimicrobial activities along with a high gut adhesion and gastrointestinal tolerance. Considering these beneficial characteristics, *L. paracasei* has potential in applications as a probiotic in biotherapy [37].

EPSs secreted by probiotics helped in the restoration of disturbed gut microflora, as it was confirmed in studies that the ingestion of active probiotic cultures contained in fermented foods, beverages, or food supplements sustains probiotic strains in the gut [38,39]. Probiotics have been used for the management of acute gastroenteritis in children [40]. Jiao et al. and other researchers have discussed the important topic of the crosstalk between gut microbiota and innate immunity and its implication in autoimmune diseases [41,42].

Table 1 presents the probiotic strains recognized for their potential to be used in the preparation of functional foods, fermented beverages, and dairy-based or non-dairy-based products and in the formulation of health supplements for their specific beneficial effects. These strains have been granted a “Qualified Presumption of Safety” (QPS) status [43,44].

## 6. Probiotic Therapy Using Biotherapeutic Agents

Probiotic strains of lactic acid bacteria (LAB) have proven their ability to synthesize many metabolites with pharmaceutical properties beneficial to health. The peptides synthesized by LAB are reported to possess anti-inflammatory activities. Bacteria secrete exopolysaccharides, which have shown antidiabetic, antioxidant, and immunomodulatory properties [11,45,46]. *Lactobacillus gasseri* is characterized as a probiotic strain and has been studied for its anti-inflammatory effects, which were demonstrated in colitis models of mice. The mechanism adopted by probiotic strain *L. gasseri* was a result of its ability to maintain the integrity of the gut barrier. Hence, the protective role of this probiotic strain could be used as a biotherapy against the progression of inflammatory intestinal diseases, IBS and IBD [47].

### 6.1. Role of Exopolysaccharides in Biotherapy Mechanisms

Probiotic strains that can stabilize in the gut are known to have beneficial effects to the host. Based on their efficiencies, various probiotic strains (Table 1) are recommended for the prevention and alleviation of several diseases [48]. They can improve metabolic activities and enable a long-lasting adjustment of the indigenous microbiota. Therefore, the improvement in the adhesion of probiotic bacterial cells in the gut is crucial for their effective colonization and maintenance of their long-term growth [49]. Strains of LAB have been reported as efficient producers of exopolysaccharides, which have shown beneficial properties in controlling the survival of gut microbiota. EPSs act as prebiotics and a source of energy, supporting the growth and colonization of gut bacteria by supplying them with nutrients [50]. The presence of EPSs in the gut also provides the necessary support for the adhesion of cells of probiotic strains in GIT to promote their long-term survival. Their adhesion is essential for the effective propagation and sustainability of a normal gut microbiota [51,52].

The synthesis of EPSs occurs through the metabolic activities of probiotic strains, which enhances the functionality of probiotics. Consequently, EPSs could be beneficial for the prevention of foodborne pathogens and for relieving symptoms of other diseases associated with pathogens. Functional natural ingredients and some organic compounds can specifically support the adhesion of bacterial strains or stimulate the expression of intestinal cell adhesion proteins. The primary role of tight junction proteins is the regulation of the intestinal barrier function, which blocks the entry of pathogenic bacteria and toxins into the vascular system [11].

The EPSs secreted by a LAB strain isolated from a dairy sample and identified as *L. paracasei* subsp. *paracasei* BGSJ2-8, have been studied for their adhesive properties to intestinal epithelial cells. Results have confirmed that the EPSs aided in the decline in *Escherichia coli*’s association with Caco-2 cells. Researchers indicated that the presence of EPSs on the surface of *Lactobacilli* might have enhanced the interaction between bacterial cells and the intestinal epithelium. This was only possible through the adhesion of probiotic cells to the gut epithelium and their effective colonization in the gut [53]. The changes in adhesion of *Lactobacillus* were studied after the bacteria was cultured in a medium containing milk supplemented with lactophospholipin. The results showed an increase in the adhesion of bacterial cells to Caco-2 cells. This biochemical activity was based on the adhesion of *Lactobacillus* promoted by the expression of related genes EF-TU and Cnb [54]. In another study by Wang et al., the physicochemical characterization and gastrointestinal adhesion of S-layer proteins-coating No I have corrected bCKliposomes were reported using a probiotic strain *Lactobacillus helveticus* [55].

### 6.2. Benefits of Probiotic Exopolysaccharides

Exopolysaccharides are important biological products produced by lactic acid bacteria. A probiotic strain of LAB, *Lactobacillus plantarum,* has been found to survive in nutritive-rich environments in the mucosae of animals and humans, and it has also been isolated from several fermented food products [56]. Its EPSs have shown several health benefits; hence, their application has been used in the food and dairy industry to exploit their capabilities to enhance the shelf-life of products. Therefore, EPSs are commercially utilized in several products to enhance the technical functionality of items [57]. In addition, EPSs support the adhesion of LAB to the epithelial lining of the human gut to facilitate the availability of nutrients to probiotic cells [58]. EPSs are also associated with the formation of biofilms and media for the adhesion of probiotic cells to gut surfaces. In biofilms, EPSs also perform many essential roles such as separating essential cations, cellular recognition, and host–pathogen interactions [59].

EPSs have been prepared from cultures of probiotic *L. plantarum*; their application showed effectiveness in improving the adhesion rate of *L. paracasei* cells to Caco-2 cells. This report was important to prove the suitability of EPSs compared to the use of other substrates as prebiotics, as similar results have been obtained from the latter. Earlier studies have indicated that only a small number of substances used as prebiotics act as a connecter between probiotic cells and the GIT lining in the host, whereas most prebiotic materials do not have a similar effect on the adhesion of probiotic cells [60,61,62]. The report recommended the benefits of EPSs, which effectively enhance the adhesion of several species of LAB. The rate of probiotic adhesion was found to be positively affected by the concentration of EPSs. This mechanism of EPS production by LAB has a definite inhibitory effect on the development of gut inflammation and the initiation of cancerous growths [63].

### 6.3. Biotherapeutic Agents Used in the Prevention of Cancer

Probiotic cultures [64] present in the gut microbiota as symbiotic partners are capable of preventing the pathogens residing in the gut by restricting their adherence to the epithelial lining; hence, they are considered live biotherapeutic agents [65]. Cell wall components were prepared from two specific yeast strains, *Kluyveromyces marxianus* and *Saccharomyces cerevisiae* var. *boulardii*. In in vitro studies, these yeast preparations showed antiproliferative, cancer chemo-preventive, and superoxide anion scavenging properties [66]. There are also reports that culture supernatants of probiotic strains contain bioactive peptides which could contribute to their antioxidant and antitumor activities; hence, the supernatants could be used for overall health benefits [31,67].

A variety of common baker’s yeast, *Saccharomyces cerevisiae*, named *S. cerevisiae* var. *boulardii*, is used as a probiotic yeast in the food and drug industries. Even though *S. boulardii* is an opportunistic pathogen, an analysis of the culture supernatant of this strain revealed different compounds in its composition, which did not show pathogenic or toxic activities. Therefore, the supernatant of this particular yeast has been recommended for its health-promoting benefits. As a therapeutic bioagent, *S. boulardii* is used to treat diarrhoea and other gastrointestinal tract disorders in neonates and adults [68]. The anticancer effects of the supernatant prepared from the culture of *S. boulardii* have been studied on cell viability, inducing apoptosis and suppression of surviving gene expression in the non-drug-resistant MCF-7 and multidrug-resistant MCF-7/MX breast cancer cells. Researchers have suggested that the *S. boulardii* supernatant can be considered as a prospective anticancer medication, along with standard treatment procedures such as chemotherapy and surgery, to treat human breast carcinoma [69]. Though more evidence through clinical data is required to validate this recommendation.

Some strains of *Escherichia coli* are part of the normal microbiota of the gut and do not cause disease in humans; such strains are harmless and even beneficial to humans. For example, non-pathogenic strains of *E. coli* help their hosts by preventing the settlement of other pathogenic bacteria in the intestine or by producing vitamin K2. These beneficial relationships between *E. coli* and humans are mutualistic biological associations [70]. *Escherichia coli* Nissle 1917 has been studied as an adaptable probiotic strain due to its long track record of not causing pathogenicity in humans. As a result of its compatibility with established techniques of genetic engineering used for bacteria, it has consequently been used as a standard starting point to obtain engineered microorganisms with therapeutic properties [71]. *E. coli* Nissle 1917 is used as a probiotic supplement for general gastrointestinal disorders, and its activity has also been assessed in randomized control trials for maintaining a reduction in ulcerative colitis [72]. Further studies have shown positive results in the treatment of inflammatory bowel disease, although with low efficacy [73]. Reports have been published where *E. coli* Nissle 1917 was used as a cellular chassis for probiotic-associated therapeutic curli hybrids. Researchers delivered matrix-tethered therapeutic domains to the gut using engineered *E. coli* Nissle 1917 [74].

The use of probiotics supports the balance of the intestinal microbiome, and a healthy gut could be an important mediator between the interaction of diet and colorectal cancer [75,76]. This was reported when a diet rich in dietary fibres with the use of whole grains was found to be associated with a lower risk of colorectal cancer, detected with positive *Fusobacterium nucleatum*. The potential role of a bacterial strain of *Bacteroides massiliensis* has been reported in the expansion of prostate cancer, as this bacterium was detected in high numbers in samples collected from patients suffering from prostate cancer [77]. Probiotics, prebiotics, synbiotics, and fermented foods act as biotics to activate a pro-carcinogenic inflammatory pathway in colonic epithelial cells [78]. The gut microbiome has also been shown to be involved in the carcinogenesis of colorectal cancer, in association with *Bacteroides fragilis*, *Fusobacterium nucleatum*, and *Peptostreptococcus anaerobius*, which are considered as potential participants in the development of colorectal cancer [79]. Therefore, the therapeutic use of probiotic cultures could support a balanced state of the gut microbiota, reduce the presence of pathogenic strains, and eliminate their involvement in the carcinogenesis of colorectal cancer [74,75,76].

### 6.4. Biotherapeutic Mechanisms in the Inhibition of Cancer Genesis

Oncogenesis or tumorigenesis is the formation of cancer cells, and it is one of the most studied pathologies associated with the gut microbiome. The link has been monitored with local gastro-intestinal cancers, as well as with other distal tumours initiated away from the point of their origin. Metabolomic and metagenomic studies have highlighted the two-fold role of the gastro-intestinal microbiome in cancer prevention and anti-cancer therapy. In effect, the different classes of bacterial strains detected in the gut microbiome can either be tumour-suppressive or oncogenic, with the potential to cause cancer. The current knowledge underlines the complexity and bi-directionality of the connection existing between the gut microbiome and the establishment of cancer. As an end result of a two-way connection, the ecology of the gut microbiome may be affected through the course of cancer development, and in its response, any changes occurring in the microbiome may affect the progression of cancer.

A number of microbe-derived compounds show anti-tumour activities, as discussed earlier; particularly, microbial-derived SCFAs may have an anti-cancer effect. Other molecules, e.g., butyrate and propionate, produced by gut bacterial strains with a general anti-cancer effect are able to inhibit the host tumour cells’ histone deacetylases. Such a mechanism was proposed to be the cause of their anti-tumoral function. The action of the butyrate effect studied in vitro and in vivo was detected in cases of colorectal cancer and lymphoma. Metabolites produced by probiotics and used as biotherapeutic agents are capable of regulating the immune system of the host. This regulation activates an indirect immune response against the initialization of cancer. For example, lipopolysaccharides can activate the signalling of TLR 4, which further stimulates the immune responses mediated by T-cells against tumour cells [80]. Some bacteria that are resident in the gut produce probiotic-derived molecules, affecting the growth of tumours. For instance, ferrichrome has been detected as a product of a probiotic strain of *L. casei* that activates the c-Jun N-terminal kinase (JNK) signalling pathway and eventually stimulates programmed cell death in cancer cells. A team of researchers confirmed that probiotic-derived ferrichrome showed inhibition of colon cancer progression via JNK-mediated apoptosis [81].

Several strains of bacteria populating the gut have been identified for their protective activity against the genesis of tumours. As a result of their capability to preserve gut homeostasis, probiotics have been assessed to help to reduce dysbiosis in patients treated with chemotherapy using different classes of antibiotics [82] and radiotherapy used for treating cancer. Three independent studies have shown that specific gut resident species may potentiate the positive outcome of anti-cancer immunotherapy. The most significant study, by Vivarelli et al., revealed the strong association between the role of the gut microbiota and tumorigenesis, together with the metabolism of gut microbiota in anti-cancer therapy [83]. Researchers have studied the function of *Lactobacillus rhamnosus* GG, the most studied probiotic model, in cancer. Based on research outcomes, novel approaches integrating the therapeutic properties of probiotics with conventional anti-cancer therapies have been strongly encouraged. Lactobacilli are also believed to boost the host immune system (DC and NK cells or TH1), which may ultimately eliminate tumour-infected cells [83].

## 7. Conclusions

The concept of biotherapy using probiotics in the form of fermented foods, probiotic beverages, probiotic dairy products, or commercial supplements has been well studied over the last decade. Other very recent studies on probiotics’ role in biotherapy have been conducted on the critical role of the gut microbiota in obesity (Cheng et al. [84]), the effects of oral glucose-lowering agents on the gut microbiota and microbial metabolites (Wang et al. [85]), the immune responses in irritable bowel syndrome (Burns et al. [86]), the role of the intestinal microbiota in non-alcoholic steatohepatitis (Xiang et al. [87]), standardizing translational microbiome studies and metagenomic analyses (Gambardella et al. [88]), the biosynthesis of gamma-aminobutyric acid, an important pharmaceutical chemical [89], the promotion of the inflammatory response by the gut microbiota in the pathogenesis of systemic lupus erythematosus (Ma et al. [90]), and the modulation of the microbiota as a therapeutic intervention for type 2 diabetes (Huda et al. [91]). These reports have specified the characterized strains of microorganisms that provide health benefits to the host by restoring or improving the gut microbiota. However, this is under the condition that they should be present in the required numbers of live cells at the time of consumption of probiotic food or supplements. We now have a better understanding of the function of the gut microbiota in an imbalanced state in the development of gut inflammation and in the pathogenesis of cancer. Therefore, balancing the gut microbiota with probiotic-based therapeutics and the beneficial properties of their metabolites, such as EPSs, has become an increasingly researched subject in the biotherapy of gut inflammation and cancer. The concluding remark is that probiotics used in biotherapy produce many health-promoting effects, such as stimulation of the host immune system, anticancer properties, and antioxidant activities.

## Figures and Tables

**Table 1:** Characterized probiotic strains with potential applications as biotherapeutic agents *.

Strains Used in Probiotic Supplements	Strains Used in Probiotic Food and Beverages
*Lactobacillus delbrueckii* subsp. *bulgaricus* *Bacillus subtilis* *Bifidobacterium bifidum* *Bifidobacterium breve* *Bifidobacterium infantis* *Bifidobacterium longum* *Lactobacillus acidophilus* *Lactobacillus casei* *Lactobacillus plantarum* *Lactobacillus rhamnosus* *Lactobacillus helveticus* *Lactobacillus salivarius* *Lactococcus lactis* subsp. *lactis* *Streptococcus thermophilus* Culture supernatant of *Probiotic yeast Saccharomyces boulardii* *Escherichia coli* Nissle 1917	*Lactiplantibacillus plantarum* *Lactococcus lactis* subsp. *cremoris* *Lactobacillus acidophilus* *Lactobacillus sporogenes* *Lactobacillus paracasei* *Lacticaseibacillus rhamnosus* *Limosilactobacillus reuteri* *Limosilactobacillus fermentum* *Levilactobacillus brevis* *Lacticaseibacillus casei* *Lactobacillus helveticus* *Streptococcus salivarius* *Kefir grains* (clusters of lactic acid bacteria and probiotic yeast)

* Information sourced from several references [26,27,31,38,39].

## Data Availability

Not applicable.

## References

[1] Saxena R., Sharma V. (2016). A Metagenomic Insight Into the Human Microbiome. Med. Health Genom..

[2] Valdes A., Walter J., Segal E., Spector T.D. (2018). Role of the gut microbiota in nutrition and health. BMJ.

[3] Yang Q., Liang Q., Balakrishnan B., Belobrajdic D.P., Feng Q.J., Zhang W. (2020). Role of dietary nutrients in the modulation of gut mi-crobiota: A narrative review. Nutrients.

[4] Kho Z.Y., Lal S.K. (2018). The Human Gut Microbiome—A Potential Controller of Wellness and Disease. Front. Microbiol..

[5] Lozupone C., Stombaugh J., Gordon J., Jansson J., Knight R. (2012). Diversity, stability and resilience of the human gut microbiota. Nature.

[6] Webb C.R., Koboziev I., Furr K.L., Grisham M.B. (2016). Protective and pro-inflammatory roles of intestinal bacteria. Pathophysiology.

[7] Boulangé C.L., Neves A.L., Chilloux J., Nicholson J.K., Dumas M.-E. (2016). Impact of the gut microbiota on inflammation, obesity, and metabolic disease. Genome Med..

[8] Sadrekarimi H., Gardanova Z.R., Bakhshesh M., Ebrahimzadeh F., Yaseri A.F., Thangavelu L., Hasanpoor Z., Zadeh F.A., Kahrizi M.S. (2022). Emerging role of human microbiome in cancer development and response to therapy: Special focus on intestinal microflora. J. Transl. Med..

[9] Mitrea L., Călinoiu L.F., Precup G., Bindea M., Rusu B., Trif M., Ferenczi L.J., Ştefănescu B.E., Vodnar D.C. (2017). Inhibitory Potential Of Lactobacillus Plantarum on Escherichia Coli. Bull. UASVM Food Sci. Technol..

[10] Hrncir T. (2022). Gut Microbiota Dysbiosis: Triggers, Consequences, Diagnostic and Therapeutic Options. Microorganisms.

[11] Cani P.D., Osto M., Geurts L., Everard A. (2012). Involvement of gut microbiota in the development of low-grade inflammation and type 2 diabetes associated with obesity. Gut Microbes.

[12] Wang Z., Shen X.-H., Feng W.-M., Ye G.-F., Qiu W., Li B. (2016). Analysis of Inflammatory Mediators in Prediabetes and Newly Diagnosed Type 2 Diabetes Patients. J. Diabetes Res..

[13] Fischbach M.A. (2018). Microbiome: Focus on Causation and Mechanism. Cell.

[14] Chung L., Orberg E.T., Geis A.L., Chan J.L., Fu K., Shields C.E.D., Dejea C.M., Fathi P., Chen J., Finard B.B. (2018). Bacteroides fragilis toxin coordinates a pro-carcinogenic inflammatory cascade via targeting of colonic epithelial cells. Cell Host Microbe.

[15] Dejea C., Wick E., Sears C.L. (2013). Bacterial oncogenesis in the colon. Futur. Microbiol..

[16] Hatakeyama M. (2017). Structure and function of Helicobacter pylori CagA, the first-identified bacterial protein involved in human cancer. Proc. Jpn. Acad. Ser. B Phys. Biol. Sci..

[17] Scott N.A., Andrusaite A., Andersen P., Lawson M., Alcon-Giner C., LeClaire C., Caim S., Le Gall G., Shaw T., Connolly J.P.R. (2018). Antibiotics induce sustained dysregulation of intestinal T cell immunity by perturbing macrophage homeostasis. Sci. Transl. Med..

[18] Woo V., Alenghat T. (2022). Epigenetic regulation by gut microbiota. Gut Microbes.

[19] Hassanpour S.H., Dehghani M. (2017). Review of cancer from perspective of molecular. J. Cancer Res. Pract..

[20] García-Castillo V., Sanhueza E., McNerney E., Onate S.A., García A. (2016). Microbiota dysbiosis: A new piece in the understanding of the carcinogenesis puzzle. J. Med. Microbiol..

[21] Zhang X., Li C., Cao W., Zhang Z. (2021). Alterations of Gastric Microbiota in Gastric Cancer and Precancerous Stages. Front. Cell Infect. Microbiol..

[22] Sun D., Chen Y., Fang J.-Y. (2019). Influence of the microbiota on epigenetics in colorectal cancer. Natl. Sci. Rev..

[23] Rezasoltani S., Asadzadeh-Aghdaei H., Nazemalhosseini-Mojarad E., Dabiri H., Ghanbari R., Zali M.R. (2017). Gut microbiota, epigenetic modification and colorectal cancer. Iran. J. Microbiol..

[24] Dahiya D., Nigam P.S. (2022). The Gut Microbiota Influenced by the Intake of Probiotics and Functional Foods with Prebiotics Can Sustain Wellness and Alleviate Certain Ailments like Gut-Inflammation and Colon-Cancer. Microorganisms.

[25] Lin C., Cai X., Zhang J., Wang W., Sheng Q., Hua H., Zhou X. (2019). Role of gut microbiota in the development and treatment of colorectal cancer. Digestion.

[26] Dahiya D., Nigam P.S. (2022). Clinical Potential of Microbial Strains, Used in Fermentation for Probiotic Food, Beverages and in Synbiotic Supplements, as Psychobiotics for Cognitive Treatment through Gut–Brain Signaling. Microorganisms.

[27] Martín R., Langella P. (2019). Emerging Health Concepts in the Probiotics Field: Streamlining the Definitions. Front. Microbiol..

[28] Matson V., Chervin C.S., Gajewski T.F. (2021). Cancer and the microbiome-influence of the commensal microbiota on cancer, im-mune responses, and immunotherapy. Gastroenterology.

[29] Huët M.A.L., Lee C.Z., Rahman S. (2022). A review on association of fungi with the development and progression of carcinogenesis in the human body. Curr. Res. Microb. Sci..

[30] Li W., Deng X., Chen T. (2021). Exploring the Modulatory Effects of Gut Microbiota in Anti-Cancer Therapy. Front. Oncol..

[31] Plaza-Diaz J., Ruiz-Ojeda F., Gil-Campos M., Gil A. (2019). Mechanisms of Action of Probiotics. Adv. Nutr..

[32] Pochapin M. (2000). The effect of probiotics on clostridium difficile diarrhea. Am. J. Gastroenterol..

[33] Tambekar D.H., Bhutada S.A. (2010). An evaluation of probiotic potential of Lactobacillus species from milk of domestic animals and commercial available probiotic preparations in prevention of enteric bacterial infections. Recent Res. Sci. Technol..

[34] Seale J., Millar M. (2013). Probiotics: A new frontier for infection control. J. Hosp. Infect..

[35] Bhatt A.P., Redinbo M.R., Bultman S.J. (2017). The role of the microbiome in cancer development and therapy. CA Cancer J. Clin..

[36] Chaurasia A.K., Thorat N.D., Tandon A., Kim J.-H., Park S.H., Kim K.K. (2016). Coupling of radiofrequency with magnetic nanoparticles treatment as an alternative physical antibacterial strategy against multiple drug resistant bacteria. Sci. Rep..

[37] Lee M.-G., Joeng H., Shin J., Kim S., Lee C., Song Y., Lee B.-H., Park H.-G., Lee T.-H., Jiang H.-H. (2022). Potential Probiotic Properties of Exopolysaccharide-Producing *Lacticaseibacillus paracasei* EPS DA-BACS and Prebiotic Activity of Its Exopolysaccharide. Microorganisms.

[38] Dahiya D., Nigam P.S. (2022). Nutrition and Health through the Use of Probiotic Strains in Fermentation to Produce Non-Dairy Functional Beverage Products Supporting Gut Microbiota. Foods.

[39] Dahiya D., Nigam P.S. (2023). Use of Characterized Microorganisms in Fermentation of Non-Dairy-Based Substrates to Produce Probiotic Food for Gut-Health and Nutrition. Fermentation.

[40] Szajewska H., Guarino A., Hojsak I., Indrio F., Kolacek S., Orel R., Salvatore S., Shamir R., Van Goudoever J.B., Vandenplas Y. (2020). Use of Probiotics for the Management of Acute Gastroenteritis in Children: An Update. J. Pediatr. Gastroenterol. Nutr..

[41] Jiao Y., Wu L., Huntington N.D., Zhang X. (2020). Crosstalk between gut microbiota and innate immunity and its implication in au-toimmune diseases. Front Immunol..

[42] De Luca F., Shoenfeld Y. (2019). The microbiome in autoimmune diseases. Clin. Exp. Immunol..

[43] Markowiak P., Śliżewska K. (2017). Effects of Probiotics, Prebiotics, and Synbiotics on Human Health. Nutrients.

[44] Herman L., Chemaly M., Cocconcelli P.S., Fernandez P., Klein G., Peixe L., Prieto M., Querol A., Suarez J.E., Sundh I. (2019). The qualified presumption of safety assessment and its role in EFSA risk evaluations: 15 years past. FEMS Microbiol. Lett..

[45] Rasmussen T.S., Koefoed A.K., Jakobsen R.R., Deng L., Castro-Mejía J.L., Brunse A., Neve H., Vogensen F.K., Nielsen D.S. (2020). Bacteriophage-mediated manipulation of the gut microbiome—Promises and presents limitations. FEMS Microbiol. Rev..

[46] Perez R.H., Zendo T., Sonomoto K. (2014). Novel bacteriocins from lactic acid bacteria (LAB): Various structures and applications. Microb. Cell Fact..

[47] Di Luccia B., Mazzoli A., Cancelliere R., Crescenzo R., Ferrandino I., Monaco A., Bucci A., Naclerio G., Iossa S., Ricca E. (2018). Lactobacillus gasseri SF1183 protects the in-testinal epithelium and prevents colitis symptoms in vivo. J. Funct. Foods.

[48] Xiao Y., Zhao J., Zhang H., Zhai Q., Chen W. (2020). Mining *Lactobacillus* and *Bifidobacterium* for organisms with long-term gut colonization potential. Clin. Nutr..

[49] De Vuyst L., Avonts L., Makras L., Remacle C., Reusens B. (2004). Probiotics, Prebiotics and Gut Health. Functional Foods, Ageing and Degenerative Dis-ease.

[50] Flint H.J., Scott K.P., Louis P., Duncan S.H. (2012). The role of the gut microbiota in nutrition and health. Nat. Rev. Gastroenterol. Hepatol..

[51] Angelin J., Kavitha M. (2020). Exopolysaccharides from probiotic bacteria and their health potential. Int. J. Biol. Macromol..

[52] Kumar A.S., Mody K., Jha B. (2007). Bacterial exopolysaccharides—A perception. J. Basic Microbiol..

[53] Živković M., Miljković M., Ruas-Madiedo P., Markelić M., Veljović K., Tolinački M., Soković S., Korać A., Golić N. (2016). EPS-SJ Exopolysaccharide Produced by the Strain Lactobacillus paracasei subsp. paracasei BGSJ2-8 is Involved in Adhesion to Epithelial Intestinal Cells and Decrease on *E. coli* Association to Caco-2 Cells. Front. Microbiol..

[54] Rocha-Mendoza D., Kosmerl E., Miyagusuku-Cruzado G., Giusti M.M., Jimenez-Flores R., Garcia-Cano I. (2020). Growth of lactic acid bacteria in milk phospholipids enhances their adhesion to Caco-2 cells. J. Dairy Sci..

[55] Wang W., Shao A., Feng S., Ding M., Luo G. (2017). Physicochemical characterization and gastrointestinal adhesion of S-layer proteins-coating liposomes. Int. J. Pharm..

[56] Mayo B., Flórez A.B., McSweeney P.L.H., McNamara J.P. (2022). Lactic Acid Bacteria: Lactobacillus plantarum. Encyclopedia of Dairy Sciences.

[57] Daba G.M., Elnahas M.O., Elkhateeb W.A. (2021). Contributions of exopolysaccharides from lactic acid bacteria as biotechnological tools in food, pharmaceutical, and medical applications. Int. J. Biol. Macromol..

[58] Russo P., López P., Capozzi V., De Palencia P.F., Dueñas M.T., Spano G., Fiocco D. (2012). Beta-Glucans Improve Growth, Viability and Colonization of Probiotic Microorganisms. Int. J. Mol. Sci..

[59] Kubota H., Senda S., Nomura N., Tokuda H., Uchiyama H. (2008). Biofilm Formation by Lactic Acid Bacteria and Resistance to Environmental Stress. J. Biosci. Bioeng..

[60] Koh J.H., Kim N., Hwang D., Lim Y.-H. (2013). Effect of water-soluble fraction of cherry tomatoes on the adhesion of probiotics and *Salmonella* to intestinal epithelial cells. J. Sci. Food Agric..

[61] Iraporda C., Rubel I.A., Manrique G.D., Abraham A.G. (2019). Influence of inulin rich carbohydrates from Jerusalem artichoke (*Helianthus tuberosus* L.) tubers on probiotic properties of Lactobacillus strains. LWT—Food Sci. Technol..

[62] Kadlec R., Jakubec M. (2014). The effect of prebiotics on adherence of probiotics. J. Dairy Sci..

[63] Wu J., Zhang Y., Ye L., Wang C. (2021). The anti-cancer effects and mechanisms of lactic acid bacteria exopolysaccharides in vitro: A review. Carbohydr. Polym..

[64] Hill C., Guarner F., Reid G., Gibson G.R., Merenstein D.J., Pot B., Morelli L., Canani R.B., Flint H.J., Salminen S. (2014). Expert consensus document: The International Scientific Association for Probiotics and Prebiotics consensus statement on the scope and appropriate use of the term probiotic. Nat. Rev. Gastroenterol. Hepatol..

[65] O’Toole P.W., Marchesi J.R., Hill C. (2017). Next-generation probiotics: The spectrum from probiotics to live biotherapeutics. Nat. Microbiol..

[66] Fortin O., Aguilar-Uscanga B., Vu K., Salmieri S., Lacroix M. (2017). Cancer Chemopreventive, Antiproliferative, and Superoxide Anion Scavenging Properties of Kluyveromyces marxianus and *Saccharomyces cerevisiae* var. boulardii Cell Wall Components. Nutr. Cancer.

[67] Thomas S., Przesdzing I., Metzke D., Schmitz J., Radbruch A., Baumgart D. (2009). Saccharomyces boulardii inhibits lipopoly-saccharide-induced activation of human dendritic cells and T cell proliferation. Clin. Exp. Immunol..

[68] Fatemi M., Ghandhari F., Karimi N. (2019). Effects of the Cell Debris and Supernatant of Saccharomyces boulardii on 7,12-Dimethylbenz(a) Anthracene-Induced Breast Cancer in Rats. J. Kermanshah Univ. Med. Sci..

[69] Pakbin B., Dibazar S.P., Allahyari S., Javadi M., Amani Z., Farasat A., Darzi S. (2022). Anticancer Properties of Probiotic Saccharomyces boulardii Supernatant on Human Breast Cancer Cells. Probiotics Antimicrob. Proteins.

[70] Martinson J.N.V., Walk S.T. (2020). *Escherichia coli* residency in the gut of healthy human adults. EcoSal Plus.

[71] Ou B., Yang Y., Tham W.L., Chen L., Guo J., Zhu G. (2016). Genetic engineering of probiotic *Escherichia coli* Nissle 1917 for clinical application. Appl. Microbiol. Biotechnol..

[72] Sonnenborn U., Schulze J. (2009). The non-pathogenic *Escherichia coli* strain Nissle 1917—Features of a versatile probiotic. Microb. Ecol. Health Dis..

[73] Scaldaferri F., Gerardi V., Mangiola F., Lopetuso L.R., Pizzoferrato M., Petito V., Papa A., Stojanovic J., Poscia A., Cammarota G. (2016). Role and mechanisms of action of *Escherichia coli* Nissle 1917 in the maintenance of remission in ulcerative colitis patients: An update. World J. Gastroenterol..

[74] Praveschotinunt P., Duraj-Thatte A.M., Gelfat I., Bahl F., Chou D.B., Joshi N.S. (2019). Engineered *E. coli* Nissle 1917 for the delivery of matrix-tethered therapeutic domains to the gut. Nat. Commun..

[75] Mehta R.S., Nishihara R., Cao Y., Song M., Mima K., Qian Z.R., Nowak J.A., Kosumi K., Hamada T., Masugi Y. (2017). Association of Dietary Patterns With Risk of Colorectal Cancer Subtypes Classified by *Fusobacterium nucleatum* in Tumor Tissue. JAMA Oncol..

[76] De Marco S., Sichetti M., Muradyan D., Piccioni M., Traina G., Pagiotti R., Pietrella D. (2018). Probiotic Cell-Free Supernatants Exhibited Anti-Inflammatory and Antioxidant Activity on Human Gut Epithelial Cells and Macrophages Stimulated with LPS. Evid.-Based Complement. Altern. Med..

[77] Golombos D.M., Ayangbesan A., O’Malley P., Lewicki P., Barlow L., Barbieri C.E., Chan C., DuLong C., Abu-Ali G., Huttenhower C. (2018). The Role of Gut Microbiome in the Pathogenesis of Prostate Cancer: A Prospective, Pilot Study. Urology.

[78] Dahiya D., Nigam P.S. (2022). Probiotics, Prebiotics, Synbiotics, and Fermented Foods as Potential Biotics in Nutrition Improving Health via Microbiome-Gut-Brain Axis. Fermentation.

[79] Wong S.H., Zhao L., Zhang X., Nakatsu G., Han J., Xu W., Xiao X., Kwong T.N.Y., Tsoi H., Wu W.K.K. (2017). Gavage of Fecal Samples from Patients with Colorectal Cancer Promotes Intestinal Carcinogenesis in Germ-Free and Conventional Mice. Gastroenterology.

[80] Sánchez-Alcoholado L., Ramos-Molina B., Otero A., Laborda-Illanes A., Ordóñez R., Medina J.A., Gómez-Millán J., Queipo-Ortuño M.I. (2020). The Role of the Gut Microbiome in Colorectal Cancer Development and Therapy Response. Cancers.

[81] Konishi H., Fujiya M., Tanaka H., Ueno N., Moriichi K., Sasajima J., Ikuta K., Akutsu H., Tanabe H., Kohgo Y. (2016). Probiotic-derived ferrichrome inhibits colon cancer progression via JNK-mediated apoptosis. Nat. Commun..

[82] Dahiya D., Nigam P.S. (2023). Antibiotic-Therapy-Induced Gut Dysbiosis Affecting Gut Microbiota—Brain Axis and Cognition: Restoration by Intake of Probiotics and Synbiotics. Int. J. Mol. Sci..

[83] Vivarelli S., Salemi R., Candido S., Falzone L., Santagati M., Stefani S., Torino F., Banna G.L., Tonini G., Libra M. (2019). Gut Microbiota and Cancer: From Pathogenesis to Therapy. Cancers.

[84] Cheng Z., Zhang L., Yang L., Chu H. (2022). The critical role of gut microbiota in obesity. Front. Endocrinol..

[85] Wang D., Liu J., Zhou L., Zhang Q., Li M., Xiao X. (2022). Effects of Oral Glucose-Lowering Agents on Gut Microbiota and Microbial Metabolites. Front. Endocrinol..

[86] Burns G.L., Talley N.J., Keely S. (2022). Immune responses in the irritable bowel syndromes: Time to consider the small intestine. BMC Med..

[87] Xiang H., Sun D., Liu X., She Z.-G., Chen Y. (2022). The Role of the Intestinal Microbiota in Nonalcoholic Steatohepatitis. Front. Endocrinol..

[88] Gambardella J., Castellanos V., Santulli G. (2021). Standardizing translational microbiome studies and metagenomic analyses. Cardiovasc. Res..

[89] Dahiya D., Manuel J.V., Nigam P.S. (2021). An Overview of Bioprocesses Employing Specifically Selected Microbial Catalysts for γ-Aminobutyric Acid Production. Microorganisms.

[90] Ma Y., Xu X., Li M., Cai J., Wei Q., Niu H. (2019). Gut microbiota promote the inflammatory response in the pathogenesis of systemic lupus erythematosus. Mol. Med..

[91] Huda M.N., Kim M., Bennett B.J. (2021). Modulating the Microbiota as a Therapeutic Intervention for Type 2 Diabetes. Front. Endocrinol..

